# Changes in the hormonal and inflammatory profile of young sprint- and endurance-trained athletes following a sports camp: a nonrandomized pretest-posttest study

**DOI:** 10.1186/s13102-024-00924-3

**Published:** 2024-06-20

**Authors:** Joanna Ostapiuk-Karolczuk, Anna Kasperska, Hanna Dziewiecka, Mirosława Cieślicka, Monika Zawadka-Kunikowska, Izabela Zaleska-Posmyk

**Affiliations:** 1 Department of Biological Sciences, Faculty of Sport Sciences in Gorzow Wielkopolski, Poznan University of Physical Education, Estkowskiego 13, Gorzów Wielkopolski, 66-400 Poland; 2 Department of Human Physiology, Nicolaus Copernicus University Ludwik Rydygier Collegium Medicum in Bydgoszcz, Karłowicza 24, Bydgoszcz, 85-092 Poland; 3 Department of Sport Theory, Faculty of Sport Sciences in Gorzow Wielkopolski, Poznan University of Physical Education, Estkowskiego 13, Gorzow Wielkopolski, 66-400 Poland

**Keywords:** Epinephrine, Norepinephrine, Cortisol, Athletes, Fatigue

## Abstract

**Background:**

The study aimed to compare catecholamine, cortisol, and immune response in sprint- and endurance-trained athletes under the same training, aiming to observe if their sport specialization affects these markers during a 9-day training camp.

**Methods:**

The study involved twenty-four young male (age 15.7 ± 1.6 years) and female (age 15.1 ± 1,3 years) athletes specializing in sprint and endurance athletics discipline. Blood samples for all measured parameters were taken at rested baseline, on the 4th day, and on the 9th day of training.

**Results:**

In both investigated groups a nonsignificant decrease in catecholamine levels was observed after 4 days of training, which remained stable throughout the camp. The cortisol level increased significantly in both athlete groups (sprint: T-0 vs. T-1 *p* = 0.0491; T-0 vs. T-3 *p* = 0.0001; endurance: T-0 vs. T-1 *p* = 0.0159; T-0 vs. T-3 *p* = 0.0005). The level of hs-CRP (sprint: T-0 vs. T-1 *p* = 0.0005; T-0 vs. T-3 *p* = 0.0001; endurance: T-0 vs. T-3 *p* = 0.0005), and myoglobin (sprint: T-0 vs. T-1 *p* = 0.0014; T-0 vs. T-3 *p* = 0.0001; endurance: T-0 vs. T-3 *p* = 0.0005) have increased and of hs-CRP and myoglobin level was significantly higher in sprint compared to endurance athletes (*p* < 0.05). The leukocyte level significantly decreased until the end of camp in both groups (sprint: T-0 vs. T-1 *p* = 0.0178; T-0 vs. T-3 *p* = 0.0175; endurance: T-0 vs. T-1 *p* = 0.0362; T-0 vs. T-3 *p* = 0.0362).

**Conclusions:**

The applied training loads had a strong physiological impact leading to changes in stress hormones and immune responses depending on athletes` sport specialization. Training loads caused stronger responses in sprint athletes. However, both groups showed signs of severe fatigue development.

**Trial registry:**

ClinicalTrials.gov ID: NCT06150105, retrospectively registered on 29.11.2023.

## Introduction

One of the essential elements of athlete training is conditioning camps, where athletes undergo a rigorous and targeted training schedule to prepare for upcoming sporting events. During training camps, the balance between training volumes and recovery is often a delicate one and the accumulation of exercise-induced stress may exceed the capacity of both neuroendocrine and immune adaptation leading to an alteration of physiological functions, decreasing adaptation of performance, immunological dysfunction, and biochemical abnormalities [1].

Physical activity acts as a powerful stimulus for the initiation of a stress hormone response involving the hypothalamic-pituitary axis (HPA) and the sympatheticoadrenal-medullary (SAM) axis. During the exercise, the adrenocortical activity demonstrates integrative functions by regulating cortisol secretion, and the sympathoadrenal by regulating catecholamines production [2]. Epinephrine (E), and norepinephrine (NE) play a significant role in the regulation of athletes’ adaptive processes. They act simultaneously on several levels allowing the realization and/or prolongation of physical exercise. Available evidence suggests that both E and NE exercise responses may be affected by several factors, such as age or training status [3].

The HPA axis also activates a longer transient hormonal cascade that terminates with the release of cortisol (C) from the adrenal cortex. It has been shown that cortisol response to exercise is related to its intensity, duration, or fitness level [3]. Earlier experimental studies have demonstrated the characteristic increase in cortisol levels after acute exercise, both in endurance- and sprint-trained athletes [3, 4]. Despite sport discipline, it appears that especially prolonged, aerobic exercise and endurance training induce the HPA axis to release large amounts of cortisol [5]. Cortisol is also used most often as a measure of stress, and a marker of fatigue or overtraining in both adolescents and adult athletes [6].

It is widely known that the HPA axis is strictly implicated also in inflammation. Its activation triggers the release of glucocorticoids, mainly cortisol, which plays a crucial role in anti-inflammatory and immunosuppressive processes [1]. Despite athletes’ discipline (sprint or endurance), significant alterations in inflammatory response after acute exercise, ranging from moderate to extremely large, were observed, but in response to a training regime, especially those with a high-training load with insufficient recovery, the prolonged increase of inflammation is observed which in consequence may lead to an excessive training-induced performance decrement [7]. According to the latest research, immune system signaling molecules, such as pro-inflammatory cytokines (i.e. interleukin-6 or tumor necrosis factor-α) secreted by immune cells such as macrophages and T-helper cells act at multiple sites within the HPA axis. Scientists suggest that this mechanism may be responsible for the negative neuroendocrine changes, observed during the experience of chronic and excessive microtrauma causing athletes to become physiologically compromised [8].

Numerous studies have evaluated changes in stress hormone levels and immune responses in adolescent athletes. However, most of these studies have focused on responses to single, intense exercises. It is well-documented that there is an increase in circulating stress hormones or inflammatory markers in response to a single exercise in young athletes, and the level of changes seems to be dependent on both exercise intensity and duration [9, 10].

However, the hormonal and immune response pattern changes when daily repeated exercises during intense training are considered. This type of research was conducted more often among adult athletes than young ones [11].

To date, very few investigations have focused on fatigue development in response to training loads in young athletes, and it is not known whether similar responses to chronic exercise are observed compared to adult ones [12, 13]. Studies concerning the influence of training on catecholamine responses in young and adult athletes are rare and remain contradictory [5, 14]. According to Armstrong et al. [15] during training with positive adaptations, cortisol levels increase in response to the stress of training. Jaheris et al. [16] have shown that just three days of intense training may elevate the cortisol level in adolescent athletes.

Moreover, prolonged exposure to repeated, high-intensity exercises (without sufficient recovery) in adolescent athletes was associated with transient immune dysfunction, elevated inflammatory biomarkers, increased risk of injury, development of fatigue, or non-functional overreaching and even overtraining syndrome [17, 18].

Endurance and sprint athletes during training develop specific adaptations in response to different training loads [19]. Moreover, cross-sectional studies have demonstrated that differences in adaptation visible as a different hormonal and immune response to exercise may exist between differently trained groups in response to a single exercise [20–22]. However, the effect of training on the stress hormones and immune responses in adolescent athletes with different sports specializations is still to be clarified.

Therefore, the purpose of the present study was to compare catecholamine, cortisol, and immune response in sprint-trained and endurance-trained athletes undergoing the same training, aiming to observe if their specialization affects these markers during a 9-day training camp.

## Methods

*Participants.* The study was a nonrandomized, time-series design with pre-and post-test comparisons. G Power v 3.1.9.7 was used to calculate the sample size for this trial *N* = 22 (11 per group) using effect size = 1.35, α = 0.05; power = 0.90 [23]. Therefore, we recruited 12 participants per group (sprint vs. endurance) in expectation of a dropout rate of 10%. Twenty-four young male and female athletes, students at Sports Championship School, future representatives of the Polish National Team, specializing in athletics disciplines such as sprint (hurdles, 100 m, 110 m), and endurance: race walk, 5000 m, and 10,000 m, volunteered to take part in this study. Inclusion criteria consisted of age 15–17 years, a minimum of 3 years of training experience, and specialization in anaerobic and aerobic disciplines. Exclusion criteria consisted of the presence of acute or chronic inflammation, fever, infections, injuries, and use of any anti-inflammatory drugs. The anthropometric measurements (Table 1) were taken a day before the camp and the body mass and composition were estimated using a bioelectrical impedance (BIA) by using Tanita Body Composition Analyzer BC-418 (Tanita Cooperation, Tokyo, Japan). Based on athletes’ specialization, they were divided into two groups: “sprint” (*n* = 12, 6 male and 6 female) and “endurance” (*n* = 12, 6 male and 6 female). The VO_2_ max was determined two weeks before the training camp using the standardized treadmill protocol.

**Table 1:** Anthropometric characteristics of participants

	Age (years)		Height (cm)	Body mass (kg)	%fat		FFM (kg)		VO_2_max (ml.kg^− 1^.min^− 1^)
**Sprint** (*n* = 12)									
Female (*n* = 6)	15.1 ± 1.3		170.9 ± 5.6	55.1 ± 5.0	21.6 ± 1.9		43.01 ± 3.4		49.1 ± 3.3
Male (*n* = 6)	15.7 ± 1.6		182.7 ± 4.8	66.9 ± 9.8	13.9 ± 2.3		58.18 ± 7.89		53.7 ± 6.9
**Endurance** (*n* = 12)									
Female (*n* = 6)	15.9 ± 1.3		165.7 ± 5.6	52.8 ± 4.0	22.2 ± 2.0		40.81 ± 2.66		55.2 ± 5.6
Male (*n* = 6)	16.6 ± 1.8		181.0 ± 13.5	63.8 ± 13.8	13.6 ± 3.0		54.15 ± 9.2		57.1 ± 8.8

Note: Data are means and standard deviation. FFM – Free-Fat Mass

*Ethical statement and clinical trial registration.* All participants and their parents were fully informed about the protocol before the start of the study and all parents gave their written consent. The study was conducted following the principles of the Declaration of Helsinki and approved by the Bioethical Committee at the Poznan University of Medical Science, Poland (Decision No. 530/18). This study adhered to CONSORT guidelines for trials and had been registered on ClinicalTrials.gov ID: NCT06150105 (retrospectively registered, on 29.11.2023).

**Fig. 1:**
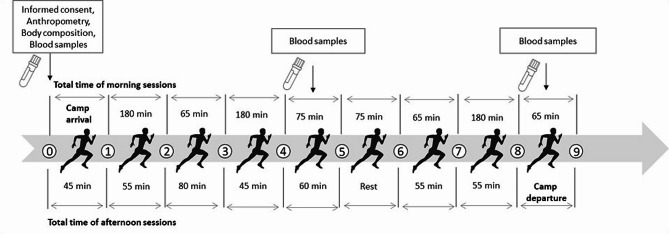
Study design and experimental protocol

*Intervention program.* The investigation was held during 9 days of the training camp (preparatory period, general preparation sub-period), which was aimed at increasing the endurance and flexibility of athletes. During the training camp, all participants resided in the same accommodations, and dietary schedules and, adhered to the same training program (the same training loads). The training schedule consisted of two training sessions per day, each lasting 4 h, for a total of 21 h over 9 days (Fig. 1). Consequently, 47% of training hours were assigned to hill tracking, 25% to agility, flexibility, and movement skills, 12% to general endurance, 10% to running strength (strength training for improve running power) and 6% was used to develop other training components, vital in athletics such as tempo running (Table 2).

**Table 2:** The training camp schedule

Days of camp	Time: 9.30–12.30 a.m.	Time: 15.00–17.00 *p*.m.
1st	Camp arrival	Jog 30`/flexibility and agility 15` Total time: 45 min
2nd	Hill tracking (hill height:1236 m) 180` Total time: 180 min	Jog 10`/flexibility and agility 45` Total time: 55 min
3rd	Jog 20`/warm-up 15`/ running strength 30` Total time: 65 min	General endurance 15`/warm-up 15`/tempo run 50` Total time: 80 min
4th	Hill tracking (hill height: 1471 m) 180` Total time: 180 min	Jog 10`/flexibility and agility 35` Total time: 45 min
5th	Warm-up 15`/strength training 60` Total time: 75 min	General endurance 15`/flexibility and agility 45` Total time: 60 min
6th	Hill tracking (hill height: 1050 m) 75` Total time: 75 min	Regeneration
7th	Jog 15`/warm-up 15`/running strength 35` Total time: 65 min	Jog 10`/agility and movement skills 45` Total time: 55 min
8th	Hill tracking (hill height: 627 m, stepping)	Agility and flexibility 10`+45` Total time: 55 min
9th	General endurance 10`/warm-up 15`/tempo runs 30` Total time: 65 min	Camp departure

*Outcome measures.* Blood samples were taken always in the same conditions, from the antecubital veins. Participants were seated in a recumbent position for a minimum of 10 min before blood draws to stabilize the hydrostatic condition. Before collection of the first sample (baseline, T-1), participants were asked to avoid any intense exercise at least 24 h before sampling. Next samples were taken after 4 days (T-2) and after another 4 days of training camp (in total after 8 days) (T-3). To avoid the effects of diurnal differences in hormone concentrations, the samples were always collected in the morning (at 7.00 a.m.), after overnight rest and fast. The hemoglobin and hematocrit, count of red and white blood cells was estimated immediately after collection (SYMEX K-4500, Poland). The blood samples for catecholamine analysis were collected using ice-cold tubes with anticoagulant and rapid centrifugated at 4^o^C to minimize the loss of catecholamines. The remaining blood samples were left at room temperature until the formation of a clot and then centrifuged at 2500 g for 10 min at 4 °C. The separated serum was transported to the laboratory within 8 h and then stored at -80 °C until analyzed.

*Primary outcomes*. The concentration of catecholamines: epinephrine and norepinephrine in serum were measured using the 2-CAT (A-N) ELISA Fast-Track kit (LDN, Germany). The intra-assay coefficient of variation (%CV) was 9.3% and 11.7% for epinephrine and norepinephrine, respectively. The concentration of cortisol, hs-CRP, and myoglobin in serum was measured using the immunodiagnostic assays: DRG Cortisol ELISA, DRG hs-CRP ELISA, and DRG Myoglobin ELISA kits (DRG International Inc., USA). The intra-assay precision (%CV) was 11%, 4,4%, and 5,4% for cortisol, hs-CRP and myoglobin, respectively. Biochemical parameters were determined using the microplate reader (Spectrostar Nano, BMG Labtech).

*Secondary outcomes*. The responsiveness of the adrenal medulla to the sympathetic nervous activity was estimated by the ratio E/NE calculated.

*Statistical analyses.* The statistical analyses were carried out with STATISTICA v. 13.0 software package (Stat–Soft, Kraków, Poland). All graphs were performed in GraphPad Prism 10.0 (GraphPad Software, Inc., San Diego, CA). Descriptive statistics, including mean and SD, were used to visualize immediate trends and patterns. All variables used in the study were checked for normality of distribution before the analyses (Shapiro-Wilk test). Levene’s test was used to verify the homogeneity of variance. Each variable depicted normal distribution, therefore one-way analysis of variance with repeated measures (ANOVA), with Tukey`s post-hoc analysis was used to assess differences in measured variables of the three assessment points (T-1, T-2, and T-3) respectively. Independent T-test was used to report the mean differences between investigated groups. Cohen`s d values were calculated by the measured variables to quantify the effect size. Using Cohen`s criteria, the effect size was interpreted as small (0.2), moderate (0.5), and large (0.8). The level of significance for all analyses was set at *p* ≤ 0.05.

## Results

The level of epinephrine in endurance-trained athletes during the whole study was slightly higher than in sprint-trained. After the first 4 days of training sessions (T-2), the level of epinephrine decreased non-significantly in both athlete groups and remained at a similar level until the end of the training camp (T-3) also, no significant differences were observed between groups at all time-points (Table 3).

**Table 3:** The effects of exercise on plasma epinephrine, norepinephrine concentration and E/NE ratio in sprint- and endurance trained athletes during sports camp

				d Cohen		
**Parameter**	**T-1**	**T-2**	**T-3**	**T-1 vs. T-2**	**T-1 vs. T-3**	**T-2 vs. T-3**
**Epinephrine** [pg.ml^− 1^]						
Sprint	565.03 ± 111.94	483.08 ± 126.35	471.29 ± 148.44	0.73	0.72	0.09
Endurance	624.51 ± 199.74	501.49 ± 127.88	502.09 ± 144.51	0.75	0.71	0.00
*p value*	0.3996	0.7573	0.6633			
**Norepinephrine** [pg.ml^− 1^]						
Sprint	1215.17 ± 407.92	1066.41 ± 311.63	1124.27 ± 215.35	0.41	0.29	0.22
Endurance	1507.24 ± 520.28	1228.23 ± 730.03	1177.77 ± 735.36	0.54	1.04	0.22
*p value*	0.1057	0.4537	0.7724			
**E/NE ratio**						
Sprint	0.43 ± 0.16	0.47 ± 0.17	0.43 ± 0.17	0.24	0.00	0.24
Endurance	0.50 ± 0.40	0.52 ± 0.26	0.69 ± 0.57	0.06	039	0.41
*p value*	0.5315	0.5624	0.1193			

Note: Data are means and standard deviation

The level of norepinephrine was also slightly higher during the study in endurance-trained athletes compared to sprint ones. After 4 days of training camp (T-2), the level of norepinephrine declined non-significantly in both investigated athlete groups and remained unchanged until the end of the camp (T-3) also, no significant differences were observed between groups at all time points (Table 3).

The level of E/NE ratio was slightly higher in endurance athletes during the study and in this group, the gradual increase of E/NE during the sport camp was observed. In sprint-trained athletes the changes in E/NE during camp were marginal. Also, no significant differences were observed between groups at all time points (Table 3).

**Fig. 2:**
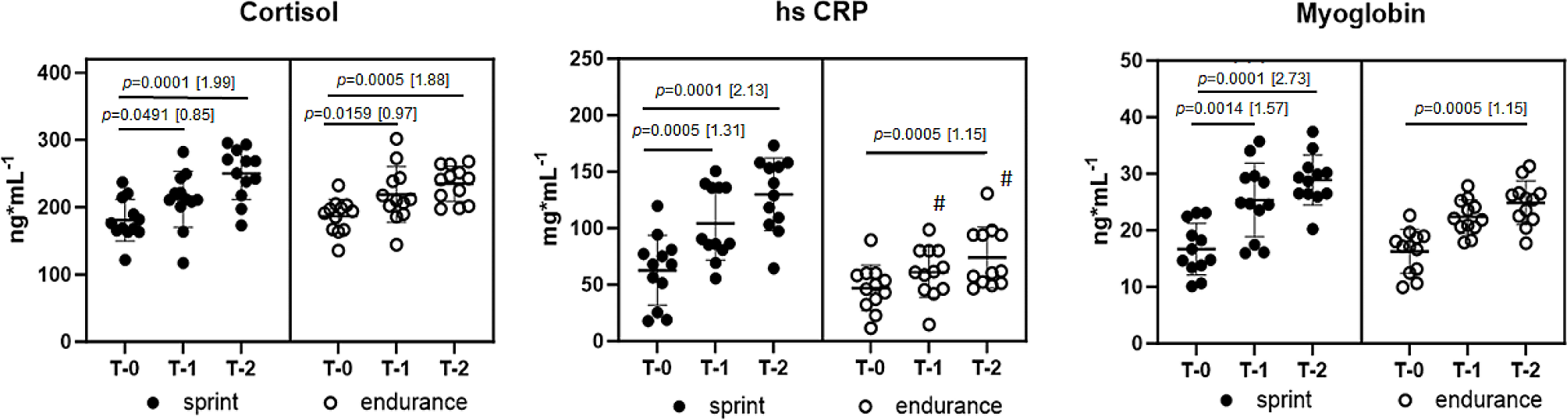
Effects of exercise on plasma cortisol, hs-CRP and myoglobin responses in sprint- and endurance-trained athletes during training camp *Note*: Data are means and standard deviation. * *p* < 0.05, ** *p* < 0.01, *** *p* < 0.001, # significant difference compared sprint to endurance at the same time points. Values in brackets represent Cohen’s d

The baseline level of cortisol was at a similar level in both sprint- and endurance-trained athletes (T-0: sprint: 176.10 ± 73.99; endurance: 203.03 ± 66.52). However, after the first 4 days of training camp, the cortisol level significantly increased in both investigated groups (T-1: sprint: 201.49 ± 50.38; T-0 vs. T-1 *p* = 0.0491; endurance: 218.85 ± 41.40; T-0 vs. T-1 *p* = 0.0159) and during the next 4 days cortisol level was still significantly growing (T-2: sprint: 242.39 ± 48.10; T-0 vs. T-2 *p* = 0.0001 endurance: 246.21 ± 3995; T-0 vs. T-2 *p* = 0.0005) (Fig. 2).

The baseline level of hs-CRP was also at a similar level in both groups (T-0: sprint: 68.98 ± 35.27; endurance: 59.23 ± 39.79), however, during the first days of the camp, the level of hs-CRP significantly increased in the sprint and endurance groups, and in the sprint group it was significantly higher than in endurance in this time point (T-1: sprint: 136.18 ± 98.63; T-0 vs. T-1 *p* = 0.0491; endurance: 79.28 ± 51.57; sprint vs. endurance: *p* = 0.0504). After another 4 days, the level of hs-CRP still significantly increased compared to the baseline level, and in the sprint group, this increase was higher than in endurance-trained athletes. Also, the level of hs-CRP in this time-point was significantly higher in the sprint- compared to endurance-trained athletes (T-2: sprint: 157.40 ± 88.52; T-0 vs. T-2 *p* = 0.0001; endurance: 93.27 ± 54.14; T-0 vs. T-2 *p* = 0.0005; sprint vs. endurance: *p* = 0.0435) (Fig. 2).

The baseline level of myoglobin was at a similar level in sprint and endurance athletes (T-0: sprint: 23.54 ± 12.10; endurance: 18.99 ± 10.02). During the first days of training camp in both investigated groups, the myoglobin level significantly increased compared to the baseline level (T-1: sprint: 24.52 ± 7.24; endurance: 21.85 ± 3.63; T-0 vs. T-1 *p* = 0.0014; endurance: 79.28 ± 51.57). After the next 4 days, myoglobin level continued to increase significantly, and in sprint-trained athletes, this growth was more rapid than in endurance one (T-2: sprint: 26.52 ± 7.91; T-0 vs. T-2 *p* = 0.0001; endurance: 24.86 ± 3.88; T-0 vs. T-2 *p* = 0.0005) (Fig. 2).

There were no significant differences in baseline level of leucocytes in athletes (T-0: sprint: 6.27 ± 1.71; endurance: 7.51 ± 3.32). After the first 4 days of training, the level of leucocytes began to significantly decrease (T-1: sprint: 5.58 ± 1.0; T-0 vs. T-1 *p* = 0.0178; endurance: 6.69 ± 2.28; T-0 vs. T-1 *p* = 0.0362) in both athletes’ groups. The statistically significant lower count of leucocytes was observed also at the end of training camp (T-3) in both investigated groups (T-2: sprint: 4.88 ± 0.68; T-0 vs. T-2 *p* = 0.0175; endurance: 5.87 ± 1.47; T-0 vs. T-2 *p* = 0.0362) (Table 4). The level of other white blood cells tested did not significantly change during the investigation (Table 4).

**Table 4:** Effects of exercise on white blood cells count sprint- and endurance-trained athletes during training camp

				d Cohen			
**Parameter**	**T-1**	**T-2**	**T-3**	**T-1 vs.** **T-2**	**T-1 vs.** **T-3**	**T-2 vs.** **T-3**	
**Leukocytes** [10^9^ x L^− 1^]							
Sprint	6.27 ± 1.71	5.58 ± 1.00^**a**^	4.88 ± 0.68^**a, b**^	0.51	1.16	0.83	
Endurance	7.51 ± 3.32	6.69 ± 2.28 ^**a**^	5.87 ± 1.47 ^**a, b**^	0.29	0.68	0.44	
*p value*	0.3314	0.2078	0.1001				
**Lymphocytes** [10^9^ x L^− 1^]							
Sprint	2.80 ± 0.67	2.31 ± 0.52	2.15 ± 0.53	0.82	1.08	0.30	
Endurance	2.66 ± 0.94	2.46 ± 0.65	2.25 ± 0.52	0.25	0.56	0.36	
*p value*	0.9639	0.9884	0.9977				
**Neutrophils** [10^9^ x L^− 1^]							
Sprint	3.00 ± 1.09	2.75 ± 0.68	2.50 ± 0.45	0.28	0.65	0.44	
Endurance	3.86 ± 2.69	3.36 ± 1.50	2.87 ± 0.49	0.24	0.62	0.4	
*p value*	0.5681	0.8476	0.9819				
**Monocytes** [10^9^ x L^− 1^]							
Sprint	0.55 ± 0.12	0.53 ± 0.11	0.51 ± 0.14	0.17	0.31	0.16	
Endurance	0.61 ± 0.18	0.58 ± 0.15	0.55 ± 0.13	0.18	0.39	0.21	
*p value*	0.8965	0.9434	0.9736				
**Eosinophils** [10^9^ x L^− 1^]							
Sprint	0.16 ± 0.10	0.16 ± 0.10	0.16 ± 0.1	0.00	0.00	0.00	
Endurance	0.28 ± 0.27	0.26 ± 0.27	0.25 ± 0.20	0.04	0.13	0.04	
*p value*	0.3301	0.4454	0.5723				
**Basophils** [10^9^ x L^− 1^]							
Sprint	0.10 ± 0.00	0.10 ± 0.00	0.10 ± 0.00	0.00	0.00	0.00	
Endurance	0.08 ± 0.05	0.08 ± 0.05	0.08 ± 0.05	0.00	0.00	0.00	
*p value*	0.5284	0.5115	0.5723				

Note: Data are means and standard deviation. a – significant differences to rest; b – significant differences to pos-exercise. Statistical significance set at *p* < 0.05

## Discussion

In our study the baseline levels of both epinephrine and norepinephrine were non-significantly higher than the subsequent measurement time points and that endurance-trained athletes had slightly higher levels of catecholamines compared to sprint-trained athletes. A similar pattern of catecholamine response was observed in experienced runners by Lehmann et al. [24]. During the experiment, they observed catecholamine response in two types of training, one with increasing training volume and a second with increasing training intensity, and results have shown a slight decrease of catecholamine levels after training with increasing volume. The dynamics of catecholamine response were also observed throughout the training season in swimmers by Hooper et al. [25]. The lower level of catecholamines was observed in seasons with intense training, compared to tapering, when catecholamines increase was observed. Most recent studies investigated hormonal responses in elite rugby players during a 6-week intense training block and a consecutive 2-week tapering period. After the training block, a significant decrease of epinephrine and norepinephrine was observed, and what is interesting, during the 2-week tapering period, catecholamines significantly increased over the pre-training values [26]. According to Barron et al. [27], excessive training volume with a lack of appropriate rest may lead to a decrease of intrinsic sympathetic activity, caused by hypothalamic dysfunction as an equivalent of central fatigue in exhausted athletes. However, results concerning catecholamine response to training are inconsistent, mainly due to different experimental protocols.

In our study, the E/NE ratio remain at the same level in sprit-trained athletes and increased in endurance-trained. Based on the evidence presented by Zouhal et al. [5], this ratio is a marker of the adrenal medulla gland’s sensitivity to sympathetic nervous stimulation. Botcazou et al. [14] reported that during the training, the adrenal medulla sensitivity increased in response to a 6-month training program in adolescent girls, similar to Abderrahman et al. [28] research, where the E/NE ratio increased in response to running interval training. According to the literature, the athletes’ sports specialization is reflected in the E/NE ratio which was higher in sprint athletes compared to non-athletes [29], and to endurance [30]. Based on the obtained results, authors presumed that short, intense exercise, such as sprints, may alter the adrenal medulla responsiveness in sprint-trained athletes, but in endurance-trained athletes, the duration of exercise may be the factor triggering the increase in the adrenal medulla sensitivity. Our study indicates that the applied training program induced fatigue-dependent changes in catecholamine responses in young, sprint-trained athletes more than in endurance-trained athletes. It also demonstrates that sport specialization (sprint or endurance) induced specific adaptations, as seen in different patterns of catecholamine response to cumulative physiological stress.

In our research, a gradual increase in cortisol was observed during the sports camp, and its level was similar in both sprint- and endurance-trained athletes. Increasing cortisol levels during periods when athletes have high training loads, are often observed in both young and adult athletes. Mishica et al. [26] observed an increase in cortisol levels in young male and female endurance-trained athletes during 7-week training after the highest training loads were applied. An increase in cortisol dependent on training load during the preparatory and competition period was also observed in female football players [31]. Also, after training based on multidirectional repeated sprints with quick changes of direction in basketball players, an increase in cortisol levels was observed. The training was aimed at improving sprinting parameters and indicated a high correlation between cortisol levels with training intensity [32]. Jürimäe et al. [33] did not observe significant changes in cortisol levels during a 6-day intensive sports camp for kayakers. However, in our study, the quick increase of cortisol in both athlete groups may indicate that applied training was too intense in the first days, causing a rapidly growing fatigue. Training loads during sports camps are often intentionally increased to obtain better preparation for the upcoming competition period. The increasing level of cortisol observed in our study is similar to observed by Minetto et al. [34], where during 10 days of overload stimuli during the training period, a gradual increase of cortisol was observed. Cadegiani and Kater [35] showed that increased cortisol may indicate an overreaching state in athletes.

According to the studies, independently of athletes’ sports specialization, in response to intense exercise, an increase in muscle damage markers and inflammation is observed. As a result of adaptive processes, the magnitude of the response is different, depending on specialization [36, 37].

In our study, an increase in hs-CRP was observed in both groups, but it was significantly higher in sprint athletes than in endurance. In both sprint- and endurance-trained athletes, a significant decline in leucocyte count was observed. Plasma myoglobin concentration also increased gradually during training and was significantly higher in the sprint group than in the endurance.

Our results are similar to those presented by Luti et al. [38], where the inflammatory response to exercises in athletes practicing sprint (soccer, basketball) was higher than in endurance sports (swimming, cycling). Souglis et al. [39] have also shown that in sports with sprint domination (soccer), muscle damage and inflammatory response to exercise were higher than in endurance activities. Also, Cipryan et al. [36] have shown that markers of muscle damage and inflammation were higher in sprint athletes compared to endurance after HIIT sessions, however, these changes were not significant. Our results may show that endurance athletes were better adapted to tolerate the training loads applied during sports camps.

Our study also showed a gradual decrease in leucocyte count, however, the changes in other white blood cell populations remained insignificant. Also, Murakami et al. [40] have observed a decline in white blood cell count in research conducted during a 9-day training camp for runners. According to Gleeson [41], the leukocyte level may decrease in response to repeated bouts of intense exercise. Research shows that the number of leukocytes gradually decreases during training and reaches low values ​​in a short time. It is now suggested that the reduction in leukocyte numbers may reflect a redistribution of cells between peripheral blood and other lymphatic compartments [42]. Moreover, the research results indicate that reducing the number of leukocytes during endurance training depends more on the increase in training volume rather than its intensity [43].

Nowadays, in endurance training, the emphasis is placed on high training intensity accompanied by a proportional high training volume. However, sprint training requires even higher training intensity than endurance, with a lower training volume [44–46]. The differences in hormonal and immune responses observed in our study seem to be the result of young athletes adapting to their disciplines. The average training experience of study participants was a minimum of 3 years. The results indicate that there is enough time to develop specific adaptations to exercise, depending on the type of training, which are reflected in a different intensity of the body’s reaction to the same training stimulus.

## Limitations

The impact of physiological stress induced by a 9-day intense training program in young, trained athletes is poorly studied. Our research has limitations, mainly the small study group, which may affect the generalization of the results. The strength of the research is that the studied groups of young athletes were already characterized by physiological adaptations to the disciplines practiced, so it was possible to observe differences in the hormonal and inflammatory responses to the same training loads. In addition, both studied groups underwent the same training protocol, which validates the comparisons among them.

## Conclusions

In conclusion, the training applied during the sports camp was characterized by a significant amount of strong physiological stimuli, the cumulative effect of which caused changes in the functioning of the HPA axis, resulting in decreased catecholamines and a significant increase in cortisol. Additionally, the study observed a decrease in leukocyte levels and an increase in inflammatory markers, which are characteristic of fatigue processes in athletes. Moreover, the young athletes’ sport specialization (aerobic vs. anaerobic) was evident in varying levels of HPA axis activity and different inflammatory responses.

Under the same training conditions, sprint-trained athletes displayed more pronounced changes than endurance-trained athletes, suggesting that endurance-trained athletes had a better tolerance for the training loads, as reflected in a weaker inflammatory response and lower cortisol levels. The results of our study highlight the importance of tailoring training programs according to athletes’ physiological responses. Monitoring changes in hormone levels, especially cortisol, can guide coaches in adjusting training loads. The results also point to the necessity of adjusting the intensity and volume of training sessions accordingly to ensure optimal performance and recovery. This approach may help prevent overtraining and mitigate fatigue-related issues. Nevertheless, further research is necessary to understand the influence of physiological stress resulting from the accumulation of training loads on HPA axis activity, not only in young but also in experienced athletes. Additionally, investigating the time course required for a complete restoration of hormonal and immune balance is crucial.

## Data Availability

The datasets generated during and analyzed during the current study are not publicly available due to confidential information about the participants but are available from the corresponding author on a reasonable request.

## Abbreviations

FFM: fat–free mass
hs: CRP–high sensitive C–reactive protein
HPA axis: hypothalamic–pituitary–adrenal axis
SAM axis: sympathetic–adreno–medullar
E: epinephrine
NE: norepinephrine
BIA: bioelectrical impedance analysis
VO_2_max: maximal oxygen consumption

## References

[1] Angeli A, Minetto M, Dovio A (2004). The overtraining syndrome in athletes: a stress-related disorder. J Endocrinol Invest.

[2] Greiwe JS, Hickner RC, Shah SD (1999). Norepinephrine response to exercise at the same relative intensity before and after endurance exercise training. J Appl Physiol (1985).

[3] Popovic B, Popovic D, Macut D (2019). Acute response to endurance Exercise stress: focus on Catabolic/anabolic Interplay between Cortisol, Testosterone, and sex hormone binding globulin in Professional athletes. J Med Biochem.

[4] Guilhem G, Hanon C, Gendreau N (2015). Salivary hormones response to Preparation and pre-competitive training of World-class level athletes. Front Physiol.

[5] Zouhal H, Jacob C, Delamarche P (2008). Catecholamines and the effects of exercise, training and gender. Sports Med.

[6] Schwarz L, Kindermann W (1990). Beta-endorphin, adrenocorticotropic hormone, cortisol and catecholamines during aerobic and anaerobic exercise. Eur J Appl Physiol Occup Physiol.

[7] Barros ES, Nascimento DC, Prestes J (2017). Acute and Chronic effects of endurance running on inflammatory markers: a systematic review. Front Physiol.

[8] Hackney AC, Lane AR (2015). Exercise and the regulation of endocrine hormones. Prog Mol Biol Transl Sci.

[9] Anderson T, Vrshek-Schallhorn S, Adams WM, Goldfarb AH, Wideman L (2023). The effect of acute exercise on the cortisol awakening response. Eur J Appl Physiol.

[10] Budde H, Machado S, Ribeiro P, Wegner M (2015). The cortisol response to exercise in young adults. Front Behav Neurosci.

[11] Nieman DC, Wentz LM (2019). The compelling link between physical activity and the body’s defense system. J Sport Health Sci.

[12] Matos N, Winsley RJ (2007). Trainability of young athletes and overtraining. J Sports Sci Med.

[13] Bank N, Hecht C, Karimi A, El-Abtah M, Huang L, Mistovich J (2022). Rising the young athlete: training and injury prevention strategies. J Pediatr Orthop Soc North Am.

[14] Armstrong LE, VanHeest JL (2002). The unknown mechanism of the overtraining syndrome: clues from depression and psychoneuroimmunology. Sports Med.

[15] Jahreis G, Kauf E, Fröhner G, Schmidt HE (1991). Influence of intensive exercise on insulin-like growth factor I, thyroid and steroid hormones in female gymnasts. Growth Regul.

[16] Botcazou M, Zouhal H, Jacob C (2006). Effect of training and detraining on catecholamine responses to sprint exercise in adolescent girls. Eur J Appl Physiol.

[17] Meeusen R, Duclos M, Foster C, Fry A, Gleeson M, Nieman D, Raglin J, Rietjens G, Steinacker J, Urhausen A (2013). European College of Sport Science; American College of Sports Medicine. Prevention, diagnosis, and treatment of the overtraining syndrome: joint consensus statement of the European College of Sport Science and the American College of Sports Medicine. Med Sci Sports Exerc.

[18] Timmons BW. (2006). *Immune Responses to Exercise in Children: A Brief Review. Pediatric Exercise Science, 18(3), 290–299*10.1123/pes.18.3.290

[19] Callister R, Shealy MJ, Fleck SJ (1988). Performance adaptations to sprint, endurance, and both modes of training. J Appl Sport Sci.

[20] Johansen L, Quistorff B (2003). 31P-MRS characterization of sprint and endurance trained athletes. Int J Sports Med.

[21] Berger NJ, Jones AM (2007). Pulmonary O2 uptake on-kinetics in sprint- and endurance-trained athletes. Appl Physiol Nutr Metab.

[22] Moll K, Gussew A, Nisser M, Derlien S, Krämer M, Reichenbach JR (2018). Comparison of metabolic adaptations between endurance- and sprint-trained athletes after an exhaustive exercise in two different calf muscles using a multi-slice ^31^ P-MR spectroscopic sequence. NMR Biomed.

[23] Kang H (2021). Sample size determination and power analysis using the G*Power software. J Educ Eval Health Prof.

[24] Lehmann M, Dickhuth HH, Schmid P (1984). Plasma catecholamines, beta-adrenergic receptors, and isoproterenol sensitivity in endurance trained and non-endurance trained volunteers. Eur J Appl Physiol Occup Physiol.

[25] Hooper SL, MacKinnon LT, Gordon RD (1993). Hormonal responses of elite swimmers to overtraining. Med Sci Sports Exerc.

[26] Mishica C, Kyröläinen H, Hynynen E (2021). Relationships between Heart Rate Variability, Sleep Duration, Cortisol and Physical Training in Young athletes. J Sports Sci Med.

[27] Barron JL, Noakes TD, Levy W (1985). Hypothalamic dysfunction in overtrained athletes. J Clin Endocrinol Metab.

[28] Ben Abderrahman A, Prioux J, Chamari K (2013). Running interval training and estimated plasma-volume variation. Int J Sports Physiol Perform.

[29] Zouhal H, Jacob C, Rannou F (2001). Effect of training status on the sympathoadrenal activity during a supramaximal exercise in human. J Sports Med Phys Fit.

[30] Zouhal H, Rannou F, Gratas-Delamarche A (1998). Adrenal medulla responsiveness to the sympathetic nervous activity in sprinters and untrained subjects during a supramaximal exercise. Int J Sports Med.

[31] Muscella A, My G, Okba S, Zangla D, Bianco A, Marsigliante S (2022). Effects of training on plasmatic cortisol and testosterone in football female referees. Physiol Rep.

[32] Brini S, Ben Abderrahman A, Boullosa D, et al. Effects of a 12-Week change-of-direction sprints Training Program on selected physical and physiological parameters in Professional Basketball Male players. Int J Environ Res Public Health. 2020;17(21). 10.3390/ijerph17218214 10.3390/ijerph17218214 PMC7664328 33172136

[33] Jürimäe J, Mäestu J, Purge P (2004). Changes in stress and recovery after heavy training in rowers. J Sci Med Sport.

[34] Minetto MA, Lanfranco F, Tibaudi A (2008). Changes in awakening cortisol response and midnight salivary cortisol are sensitive markers of strenuous training-induced fatigue. J Endocrinol Invest.

[35] Cadegiani FA, Kater CE (2019). Enhancement of hypothalamic-pituitary activity in male athletes: evidence of a novel hormonal mechanism of physical conditioning. BMC Endocr Disord.

[36] Cipryan L, Tschakert G, Hofmann P (2017). Acute and post-exercise physiological responses to high-intensity interval training in endurance and Sprint athletes. J Sports Sci Med.

[37] Mohr M, Draganidis D, Chatzinikolaou A, Barbero-Álvarez JC, Castagna C, Douroudos I, Avloniti A, Margeli A, Papassotiriou I, Flouris AD, Jamurtas AZ, Krustrup P, Fatouros IG (2016). Muscle damage, inflammatory, immune and performance responses to three football games in 1 week in competitive male players. Eur J Appl Physiol.

[38] Luti S, Modesti A, Modesti PA (2020). Inflammation, peripheral signals and Redox Homeostasis in athletes who practice different sports. Antioxid (Basel).

[39] Souglis AG, Chryssanthopoulos CI, Travlos AK, Zorzou AE, Gissis IT, Papadopoulos CN, Sotiropoulos AA (2013). The effect of high vs. low carbohydrate diets on distances covered in soccer. J Strength Cond Res.

[40] Murakami S, Kurihara S, Titchenal CA, Ohtani M. Suppression of exercise-induced neutrophilia and lymphopenia in athletes by cystine/theanine intake: a randomized, double-blind, placebo-controlled trial. *J Int Soc Sports Nutr* 2010; 4;7(1):23. 10.1186/1550-2783-7-23 10.1186/1550-2783-7-23 PMC2892463 20525371

[41] Gleeson M (2002). Biochemical and immunological markers of over-training. J Sports Sci Med.

[42] Gunzer W, Konrad M, Pail E (2012). Exercise-induced immunodepression in endurance athletes and nutritional intervention with carbohydrate, protein and fat-what is possible, what is not?. Nutrients.

[43] MacKinnon LT, Special feature for the Olympics: effects of exercise on the immune system: overtraining effects on immunity and performance in athletes. 2000; 78(5):502-9. 10.1111/j.1440-1711.2000.t01-7-.x. Gunzer W, Konrad M, Pail E. Exercise-induced immunodepression in endurance athletes and nutritional intervention with carbohydrate, protein and fat-what is possible, what is not? *Nutrients* 2012; 4(9):1187–1212. Doi: 10.3390/nu4091187. 10.3390/nu4091187 PMC3475230 23112908

[44] Lehmann M, Mann H, Gastmann U (1996). Unaccustomed high-mileage vs intensity training-related changes in performance and serum amino acid levels. Int J Sports Med.

[45] Skovgaard C, Christiansen D, Christensen PM, Almquist NW, Thomassen M, Bangsbo J (2018). Effect of speed endurance training and reduced training volume on running economy and single muscle fiber adaptations in trained runners. Physiol Rep.

[46] Bangsbo J (2015). Performance in sports-with specific emphasis on the effect of intensified training. Scand J Med Sci Sports.

